# Integrating dispersal, breeding and abundance data with graph theory for the characterization and management of functional connectivity in amphibian pondscapes

**DOI:** 10.1007/s10980-022-01520-x

**Published:** 2022-11-03

**Authors:** Ismael Reyes-Moya, Gregorio Sánchez-Montes, Íñigo Martínez-Solano

**Affiliations:** Departamento de Biodiversidad y Biología Evolutiva, Museo Nacional de Ciencias Naturales (MNCN-CSIC), c/ José Gutiérrez Abascal 2, 28006 Madrid, Spain

**Keywords:** Graph theory, Pond-breeding amphibians, Connectivity, Dispersal, Photo-identification, Mark–recapture

## Abstract

**Context:**

Robust assessment of functional connectivity in amphibian population networks is essential to address their global decline. The potential of graph theory to characterize connectivity among amphibian populations has already been confirmed, but the movement data on which modelled graphs rely are often scarce and inaccurate. While probabilistic methods that account for intraspecific variability in dispersal better reflect the biological reality of functional connectivity, they must be informed by systematically recorded individual movement data, which are difficult to obtain for secretive taxa like amphibians.

**Objectives:**

Our aim is to assess the applied potential of probabilistic graph theory to characterize overall connectivity across amphibian pondscapes using fine-scale capture-recapture data, and to inform conservation management based on the role of ponds on functional connectivity.

**Methods:**

We monitored an amphibian community in a pondscape located in a Spanish “dehesa” for 2 years. Photoidentification was used to build capture histories for individuals of six species, from which dispersal kernels and population sizes were estimated to model probabilistic graphs.

**Results:**

We obtained kernels of variable robustness for six species. Node importance for connectivity varied between species, but with common patterns such as shared road crossing areas and the presence of coincident interconnected pond clusters.

**Conclusions:**

The combination of photoidentification, capture-recapture data and graph theory allowed us to characterize functional connectivity across the pondscape of study accounting for dispersal variability and identify areas where conservation actions could be most efficient. Our results highlight the need to account for interspecific differences in the study and management of amphibian pondscapes.

**Supplementary Information:**

The online version contains supplementary material available at 10.1007/s10980-022-01520-x.

## Introduction

Amphibians are the most endangered vertebrate group on the planet, with 41% of their species listed as threatened (Catenazzi 2015; IUCN 2021). Habitat destruction is one of the main factors involved in worldwide amphibian declines. Loss of entire patches of habitat leads not only to decreased overall population sizes but also to reduced functional connectivity among surviving demes, collectively raising their extinction risk (Burkey 1989; Fahrig 1997; Stuart et al. 2004; Fischer and Lindenmayer 2007; Zamberletti et al. 2018). Given the importance of addressing habitat loss and fragmentation in amphibian conservation, efforts have been undertaken to promote population connectivity, notably through the construction of artificial wetlands which may serve as stepping-stones (Lesbarrères et al. 2010; Ruhí et al. 2012).

The robust characterization of patterns of functional connectivity, i.e. the movement of species among resource patches, is cornerstone for the effective design of these conservation measures (Vogt et al. 2009; Braaker et al. 2014). Functional connectivity is fundamental to characterize (meta)population dynamics, because it conditions local rescue, colonization and extinction probabilities, and is a prerequisite for the persistence of “sink” populations (Levins 1969; Taylor et al. 1993; Figueira and Crowder 2006; Vogt et al. 2009). Functional connectivity is driven by dispersal, which is the unidirectional movement of individuals or propagules across space with potential consequences for gene flow (Ronce 2007). The importance of dispersal in ecology and evolutionary biology has led to the development and application of direct and indirect approaches for its study (Broquet and Petit 2009; Cayuela et al. 2018). However, while dispersal events have been comparatively well documented in mammals (Woodroffe et al. 1995; Zimmermann et al. 2005), birds (Fernandes et al. 2014; Kingma et al. 2017) or some reptiles (Tucker et al. 1998; Dubey et al. 2008), large knowledge gaps remain for amphibians (Cayuela et al. 2020). Amphibian dispersal comprises movements between breeding ponds (breeding dispersal) as well as the movement of juveniles from their birth pond to a different reproductive pond (natal dispersal) (Cayuela et al. 2020). After reproduction, some amphibians leave the reproductive ponds to move to their terrestrial habitat, from which they will return in the next breeding season; these repeated movements are considered migration rather than dispersal (Semlitsch 2008).

Functional connectivity in amphibian population dynamics has been assessed with different approaches, including the characterization of genetic structure at the landscape scale (Richardson 2012; Watts et al. 2015), occupancy models (Brooks et al. 2019), electric circuit theory (Beaujean et al. 2021; Costa et al. 2021), and graph theory (Ribeiro et al. 2011; Matos et al. 2019; Schivo et al. 2020). The latter has been successfully applied for the study of patterns of connectivity in fragmented landscapes (Bunn et al. 2000; Jordán et al. 2003, 2007; Bodin and Norberg 2007; Vasas et al. 2009), and is a promising tool for characterizing the structure and dynamics of pond-breeding amphibian metapopulations. A graph is the mathematical formalization of a network of data points, interchangeably termed nodes or vertices, which are connected by some kind of relationship. These connections are indistinctly called edges or links. In population ecology, nodes usually correspond to habitat patches, while edges represent a flux, such as dispersal between nodes (Urban and Keitt 2001). For pondscapes, nodes can represent the ponds where amphibians congregate for reproduction and, when available, quantitative attributes such as demographic data (like abundance or breeding success) can be used to weight their respective importance. However, modelling population graphs is usually dependent on the threshold defining whether or not two nodes are connected. This threshold is usually set as the maximum dispersal distance recorded for the study species: if the distance between two nodes is lower than the threshold distance used, the two nodes are considered connected. In some studies, distance is weighted by the landscape resistance (see Bunn et al. 2000; Decout et al. 2012; Matos et al. 2019).

Unfortunately, amphibian dispersal data are scarce, as dispersal in this group is relatively infrequent (Cayuela et al. 2020) and thus, difficult to detect. When available, dispersal data are often based on opportunistic or anecdotal records and thus probably inaccurate or non-representative. Additionally, connectivity studies are often conducted at geographical scales that are much larger than the typical dispersal distances of amphibians (Sinsch 2012). Therefore, when modelling graphs, researchers must rely on sequential thresholds (Fortuna et al. 2006) or records of maximum dispersal from other studies, often obtained from different geographic areas (Ribeiro et al. 2011; Clauzel et al. 2013) or from different species (Schivo et al. 2020). This approach is limited and prone to biases of unknown magnitude due to the high interspecific variation of dispersal distances described in amphibians, even among species from the same family (Cayuela et al. 2020). Furthermore, there is evidence for intrapopulation variation in dispersal distances in pond-breeding amphibians, with a small fraction of the individuals performing rare long-distance dispersal events while much more frequent, short distance movements are carried out by a higher proportion of the population (Sánchez-Montes et al. 2018; Cayuela et al. 2020).

To account for the intraspecific variability in dispersal frequencies and distances, dispersal can be regarded as a probabilistic variable rather than a fixed maximum value when designing a graph (Sullivan et al. 2021). This method is more biologically realistic, relying on the use of dispersal kernels to estimate dispersal probabilities. Nevertheless, the accurate modelling of dispersal kernels requires a substantial number of direct observations of displacement events, which are rarely available for amphibians. Given these limitations, reconstructing robust and biologically realistic amphibian dispersal graphs remains challenging, thereby seriously compromising conservation efforts.

We applied a probabilistic approach based on graph theory to a systematically recorded empirical dataset to model fine-scale connectivity graphs and quantify the contribution of each pond to functional connectivity in an amphibian community. Specifically, we recorded information on abundance, breeding activity and fine-scale individual dispersal events based on photoidentification capture-recapture data in an amphibian pondscape with a high density of temporary ponds over two consecutive breeding seasons. Based on these data, we aimed to: (1) generate dispersal kernels based on actual movement data from syntopic amphibian species, (2) construct probabilistic graphs to provide quantitative assessments of functional connectivity for each species across the pondscape, and (3) assess the leverage of each pond in the connectivity of the whole graph. To our knowledge, this is the first study using fine-scale field estimates of dispersal based on capture-recapture data to model species-specific connectivity graphs for pond-breeding amphibian communities. Our results demonstrate the suitability of this method for the characterization of fine-scale amphibian pondscapes, allowing species-specific, biologically-informed prioritization of ponds for amphibian conservation based on their role on functional connectivity. This method also allows identification of key areas that could benefit from specific management actions aimed at enhancing population connectivity.

## Materials and methods

### Study area and sampling

**Fig. 1:**
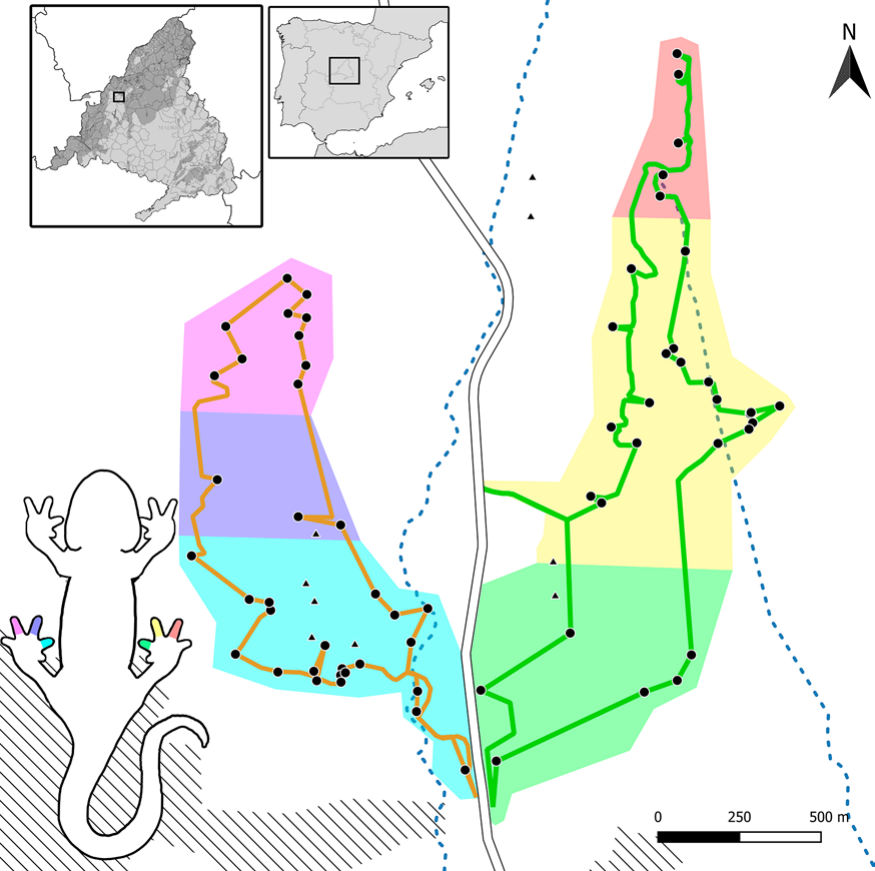
Map of the study area showing predefined sampling subsectors and the corresponding toe-clipping code for urodeles (six colors), monitored ponds (black dots), unsampled ponds (triangles), approximate transect paths (colored lines), the M601 road (white line), temporary streams (blue dashed lines) and urban areas (black dashed areas). The inset maps show the location of the study area at the west of the Community of Madrid (central Spain)

The study pondscape is located in a holm oak (*Quercus ilex*) dehesa in the municipality of Alpedrete (Madrid, central Spain). The area is divided by road M601 (running North-South with a Daily Average Intensity of 15,735 vehicles, Ayuntamiento de Collado Mediano 2018) into western (with 33 temporary ponds) and eastern (30 temporary ponds) sectors. For the purposes of this study, each sector was further subdivided into three sampling subsectors (northern, central and southern, Fig. 1), which were comprehensively surveyed during the flooding hydroperiods of the temporary ponds in 2019 and 2020. In 2019, the sampling season extended from February 13th to September 29th, with additional sampling from the 10th to the 15th of November. In 2020, sampling started on January 9th and extended to July 23rd. Sampling was halted between March and April of 2020 due to mobility restrictions enforced during the COVID-19 outbreak.

We monitored a total of 64 ponds, 63 temporary (drying completely every year) and one permanent. Seven large deep permanent ponds with a high density of predatory invasive fish and crayfish were excluded from the study because they are unsuitable to sustain viable amphibian populations (Fig. 1). During the first year, all ponds of both sectors were sampled once a week. The second year, precipitation was more abundant and the number of captured individuals per pond increased, thus requiring higher sampling effort per pond, which led us to sample all ponds biweekly. Sampling was performed one sector at a time, and consisted of standardized transects surveyed on foot, covering a total of 6.2 and 6.7 Km in the western and eastern sectors, respectively (Fig. 1). All ponds in the same sector were sampled in a single night per week during 2019 and in two nights biweekly in 2020, and sampling sessions occurred just after or during rainfall, where possible. Sampling started after dusk (around 8 pm in winter and 10 pm in summer), to coincide with the nocturnal habits of amphibian species in the area and lasted until all ponds were sampled (a maximum of 11 h) or until dawn (around 7 am in winter and 5 am in summer). If sampling could not be completed before dawn, an additional sampling night was scheduled in the same week. Finally, three complementary sampling sessions were performed during the 2021 breeding season (from April 15th to June 12th ) to sample all 64 ponds and detect potential additional dispersal events of animals marked in 2019 and 2020.

In each sampling session, we aimed to detect and individually identify all sexually mature amphibians in the sampling areas. The amphibian community inhabiting the study area is composed of 10 species, including 3 urodeles: *Pleurodeles waltl* Michahelles 1830, *Triturus pygmaeus* (Wolterstorff 1905) and *Lissotriton boscai* (Lataste *in* Tourneville 1879); and 7 anurans: *Bufo spinosus* Daudin 1803, *Epidalea calamita* (Laurenti 1768), *Pelophylax perezi* (López-Seoane 1885), *Hyla molleri* Bedriaga 1889, *Pelobates cultripes* (Cuvier 1829), *Discoglossus galganoi* Capula, Nascetti, Lanza, Bullini & Crespo, 1985 and *Alytes cisternasii* Boscá 1879. Each sampling session was carried out by a team of 2–5 researchers, who exhaustively surveyed each pond and a corresponding terrestrial transect and captured sexually mature individuals using baited fish traps (trapping was performed in 3–4 intervals of 20 min in each of the four deepest ponds, targeting urodeles), dip nets or by hand.

We identified the species and sex of each captured animal and recorded the capture location with a Global Positioning System (Garmin eTrex 30x, precision: 3.65 m). For our analyses, animals captured within ponds or within 10 m of the shore were associated with the geographical coordinates of the corresponding pond, whereas animals found in terrestrial habitat along standardized tracks were georeferenced individually. We clipped three phalanges of a single toe of each individual for future genetic and skeletochronological studies, and also to identify the approximate marking location of individuals: we clipped a different toe for animals captured in different sectors and subsectors to aid photoidentification. For this purpose, we clipped the 4th toe (usually the longest) of the left hindlimb of anurans first captured in the west sector and the 4th toe of the right hindlimb in individuals first captured in the east sector. In urodeles, in addition to this right-left distinction, we clipped the 2nd, 3rd, or 4th toe depending on whether they were found in the southern, central or northern subsector, respectively (Fig. 1). For individuals recaptured in a different (sub)sector from their marking place, we clipped an additional toe corresponding to the new (sub)sector, but no individual required more than two toes clipped, which has been shown to have a negligible effect on survival in amphibians (McCarthy and Parris 2004).

At capture, we took photographs of all adult individuals with different devices (cell phones, digital cameras) depending on availability (resolution from 72 to 300 ppp). For anurans, photos were taken perpendicularly to the dorsal surface of the animal in a natural resting position and illuminated from the top with a neutral white light. We took two photos of each animal, with and without flash, to control for illumination artifacts. For *E. calamita*, as their skin was not reflective, a single photo was taken without flash to reduce handling time. For *H. molleri*, we took a third photo in lateral view so that the contour of the black lateral band, a variable feature in this species, could be recognized (Online Resource 1: Fig. S1). Animals found along the terrestrial transects were rinsed with water before taking the shots to facilitate subsequent pattern recognition.

Urodele photographs were taken following Mettouris et al. (2016), where animals were deposited in a transparent plastic tray and immobilized in a straight position with the aid of a soft sponge so that the belly of the animal could be photographed (Online Resource 1: Fig. S1). Photos of urodeles were taken without flash. Due to sampling difficulties, photographs of urodeles were only taken toward the end of the first year’s field season. Thus, our capture histories are shorter for urodeles than for anurans, as not all recaptured individuals are photoidentified (Table 1). All animals were released at their place of capture immediately after processing. We also identified and recorded larvae and egg clutches in all sampled ponds to gather a comprehensive list of the breeding ponds used by each species.

### Photo-identification

We used the photographs obtained in the field for individual identification. Most of this process was performed manually, but we used the software program Aphis (Moya et al. 2015) to aid manual identification of some *E. calamita* individuals using the I3S analysis. For this species, we used the lateral canthi of the eyes and the distal end of the urostyle as reference landmarks and the center of the warts as matching landmarks, selecting those closer to the longitudinal axis of the toad and on top of the head, so that the distance between them was less affected by the changes of position of the animal. However, Aphis had problems identifying individuals with a large density of uniformly spaced warts. Thus, most *E. calamita* were identified manually. WildID (Bolger et al. 2012) was used to aid in the identification of individual *P. perezi*, *P. waltl, T. pygmaeus*, and *L. boscai*, and to check for possible errors during manual identification.

### Abundance and dispersal

The identification of individuals in each sampling session allowed us to compile individual capture histories, which were used to obtain a single population abundance estimate at each pond using the combined data from 2019 to 2020 and model the dispersal kernels for each species. In ponds with very few captures, or in large populations with few recaptures (as in many *E. calamita* sites), we used the total number of individuals photoidentified as a proxy for population size. Although underestimated, these population size estimates are comparable among the ponds of our study area, since all ponds of each sector were sampled very close in time (often in the same night). In ponds where a sufficient proportion of recaptures were obtained (minimum proportion of individuals recaptured at least once = 0.08), we ran capture-recapture analyses to estimate population sizes using POPAN models (Schwarz and Arnason 1996) implemented in RMark (Laake 2013). The POPAN formulation consists on a combination of four different parameters: apparent survival probability (*Φ*), capture probability (*p*), probability of entrance of individuals in the population (*pent)*, and population size (*N*), which are altogether estimated in a maximum-likelihood approach with respect to the observed capture histories (i.e., the data). The POPAN parameters can be modeled as constant or variable across different groups of individuals (e.g., males vs. females) or sampling sessions (i.e., time), thus producing a set of linear predictors. The likelihood of each set of linear predictors is then calculated with respect to the response variable, which ultimately corresponds to whether each animal was either seen or not in a given sampling session. We used capture history data from 2019 to 2020, excluding sessions with a low number of captures to avoid model overparameterization. Goodness of fit (GOF) testing was performed with r2Ucare to assess the adjustment of the dataset to POPAN assumptions (Gimenez et al. 2018).

We used stepwise model selection in rMark, starting with a fully parameterized model with *Φ*, *p* and *pent* as dependent on time, sex and their interaction. This saturated model was compared to three other models, which were identical except for *Φ* being either constant, time-dependent or sex-dependent. The best of the four initial candidate models was then selected and *Φ* was accordingly fixed, so that in further steps different parameterizations of *p* and then *pent* were tested using the same procedure. A *pent* constant through time was *a priori* discarded from the biologically meaningful set of candidate models, based on documented amphibian population dynamics and our own field observations (species in our study area are not equally active throughout the year, but have well-delimited breeding seasons during which most adult individuals congregate in ponds, see for instance García-París et al. 1989). We obtained abundance estimates for males and females of each species by averaging *N* estimates from the candidate models, weighted by their AICc (Akaike 1973) or using the best fitting model if AICc weight was close to one.

To obtain dispersal kernels for each species, we plotted density histograms of the movement distances recorded in the field. These histograms were then fitted with lognormal and Weibull distributions, both fat-tailed distributions, as recommended for dispersal kernels by Nathan et al. (2012). The distribution with the lowest AIC value was selected as the best fitting distribution. To detect possible differences in the average dispersal distance between species, we performed a Tukey test (α = 0.05) with the raw dispersal distance data from all species. Movement frequencies were also calculated as the proportion of recaptures between 2019 and 2020 for which movement was detected.

### Pondscape graph modeling

**Fig. 2:**
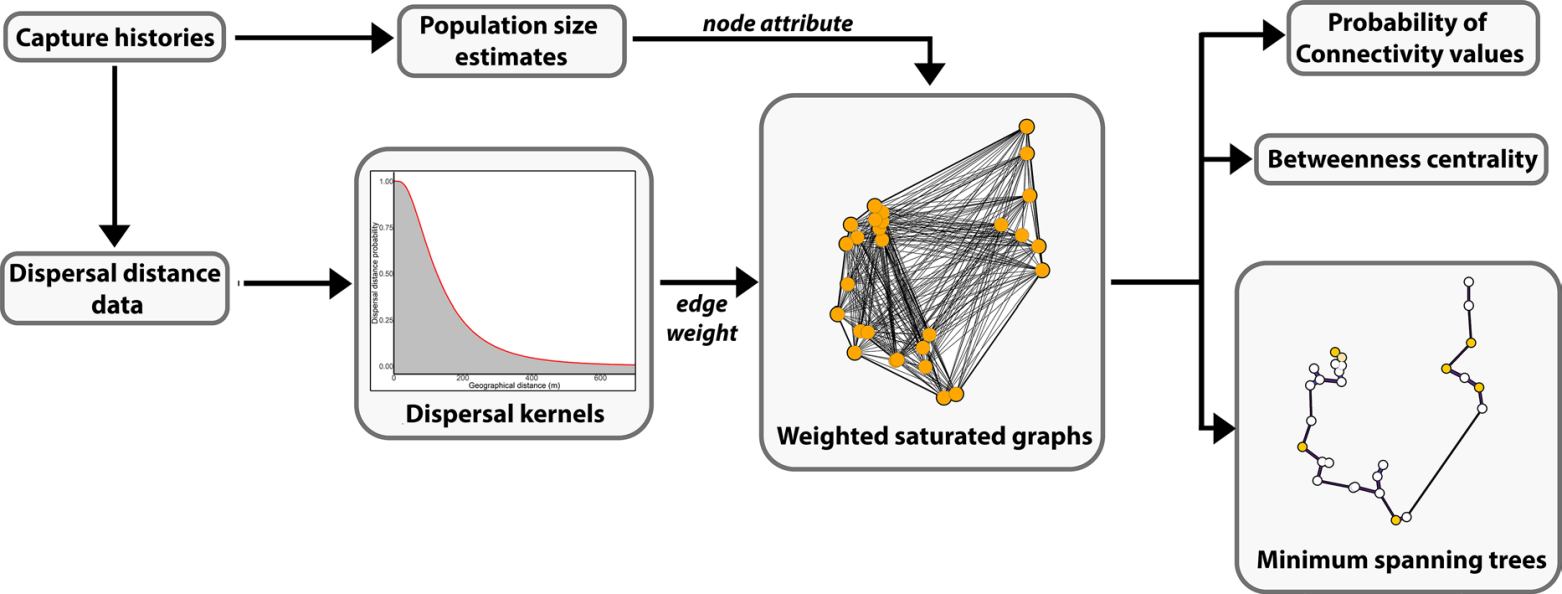
Workflow of the main steps and data sources of the study. Individual capture histories are used to detect movements and estimate population sizes, which are in turn used as node attributes (variables attached to nodes). Dispersal distances are fitted to a distribution to obtain dispersal kernels, which are then used to weight the edges according to their Euclidean distance, as a proxy for dispersal probability. Weighted graphs are finally used to calculate Probability of Connectivity values, betweenness centrality and minimum spanning trees

To characterize the connectivity of the pondscape, we generated a saturated graph (i.e., presenting all possible connections) for each species using igraph (Csardi and Nepusz 2006) with ponds as nodes and their direct connections as edges. Edge weight was defined as the probability, based on the species specific dispersal kernel, that an animal would travel the Euclidean geographical distance between the two nodes of each edge (Fig. 2). To account for the effect of the road as a barrier to amphibian movement (Gibbs 1998; Jochimsen et al. 2004; García-González et al. 2012), we calculated road resistance as twice the geographical distance between nodes located on opposite sides of the road. While other options for including road resistance were tested, all of them resulted in the southern part of the study site being identified as the optimal area for road crossing, given the long distances separating the nearest ponds from opposite sides of the road in the northern part of the study area.

To characterize the contribution of each pond to the metapopulation functional connectivity, we calculated the probability of connectivity of the whole graph (PC) and the variation of this metric when each node *k* was removed (dPC_k_), as described by Saura and Rubio (2010). To achieve this, for each of the species we defined the estimated population size of the ponds as the node attributes (a) and the total population size of the network (the sum of all node attributes) as the maximum landscape attribute (A_L_).

The parameter dPC_k_ can be further decomposed into three fractions (dPCintra_k_, dPCflux_k_ and dPCconnector_k_), which were also calculated to obtain a more specific assessment of the role of each pond in functional connectivity. First, dPCintra_k_ refers to the contribution of node *k* to *A*_*L*_ in terms of its estimated population size. Therefore, this fraction depends solely on node attributes, i.e., nodes with high population sizes have high dPCintra_k_ values regardless of their connectivity. Second, dPCflux_k_ describes the connectivity of node *k* to the rest of nodes of the graph, weighing their shortest path product probability by the population sizes at both ends. This parameter provides a proxy for (1) how well-connected node *k* is, and (2) the potential amount of individuals using these connections. Therefore, nodes with high population sizes that are well connected to other populous nodes have a high dPCflux_k_ value. Finally, dPCconnector_k_ evaluates the contribution of node *k* to the connectivity as an intermediate stepping-stone inside shortest paths.

For PC related metrics, path length is computed as the product probability of the edges of a path (Saura and Rubio 2010). The shortest path between a pair of nodes is therefore defined as the path with the maximum product probability of all possible paths between those two nodes. This multiplicative method penalizes the use of stepping-stones in long distance movements when compared to summative methods: given the asymptotic characteristics of the kernels, if three aligned nodes are separated by small connection probabilities, the probability of the direct path between the nodes located at the extremes will be higher than the product probability of the path that uses the stepping-stone between them. Stepping-stones are nevertheless of key importance for long distance dispersal (Baum et al. 2004; Saura et al. 2014), as small patches of favorable habitat can serve as temporary refugia for amphibians during displacement (Chan-Mcleod and Moy, 2007), sheltering them from the more unfavorable matrix in which they can suffer an increased risk of dehydration and predation. Thus, to identify ponds that could be acting as stepping-stones in long distance dispersal movements, we calculated betweenness centrality for each node (Anthonisse 1971; Freeman 1977), a measurement of the number of shortest paths that use a node as stepping-stone. For these calculations we defined edge weight as the inverse of the connection probability and path length as a sum of these weights instead of a product.

To obtain a measurement of fragmentation of the graph we used a walktrap clustering function (Pons and Latapy 2005) as implemented in igraph and compared the number of clusters recovered for each species. These clusters represent groups of highly connected nodes that are loosely connected to the rest of the graph.

Complementarily, we built movement graphs to illustrate the detected movements using the number of movements as edge weights (Online Resource 1: Fig. S2). We also built Minimum Spanning Trees (MSTs) to show the most likely connections between clusters (Online Resource 1: Fig. S3). MSTs are subsets of the saturated graph, consisting of simplified networks that connect all nodes while keeping the total sum of edge weights to a minimum and without any loop (paths that start and end at the same node).

We obtained movement data for only a subset of the amphibian species in the study area, but even for those without movement data, we obtained abundance estimates or, at least, a list of all reproductive ponds used. Because management actions in this pondscape would benefit from this additional information concerning rarer species, we created a simple complementary index taking into account species richness and abundance estimates, to measure the importance of each pond for the populations of all 10 species combined. To achieve this, we scaled the population estimates for each species in all ponds and summed the scaled values of all species for each pond. The resulting index assesses the importance of each pond in relation to its contribution to the total amphibian community.

## Results

### Captures and recorded dispersal

We marked 2249 total individuals in 2019 and 2287 in 2020. The number of recaptures were 1529 in 2019, of which 933 were photoidentified, and 1022 in 2020 (recaptures from 2020 also included surviving individuals marked in 2019), all of which were photoidentified. No adult *A. cisternasii* individuals were found, and their presence was only recorded from calls, tadpoles and froglets. Color changes were detected for several species (Online Resource 2). A summary of capture statistics per species is shown in Table 1.

We detected differences in the distribution of some species on opposite sides of the road (Table 1, Online Resource 1: Figs. S4 and S5). For instance, captures of *P. perezi* and *T. pygmaeus* were more numerous in the western sector, while *L. boscai* captures were mainly obtained in the eastern sector. Some species were completely absent from one of the sectors. *Hyla molleri* and *D. galganoi* were absent from the eastern sector, and *B. spinosus* and *A. cisternasi* were not detected in the western sector. *Discoglossus galganoi* and *A. cisternasii* were also very rare and restricted to very small areas.

We detected a total of 213 individual dispersal events for 6 of the 10 species present in the study area. A single marked individual of *P. cultripes* was detected having crossed the road dividing both sectors. Detailed dispersal records are shown in Table 1, and maps showing detected movements for each species are provided in Online Resource 1: Figure S2. Only the differences between average movements of *E. calamita* and *P. perezi* were significant (Tukey test, *p*-value = 0.023).

**Fig. 3:**
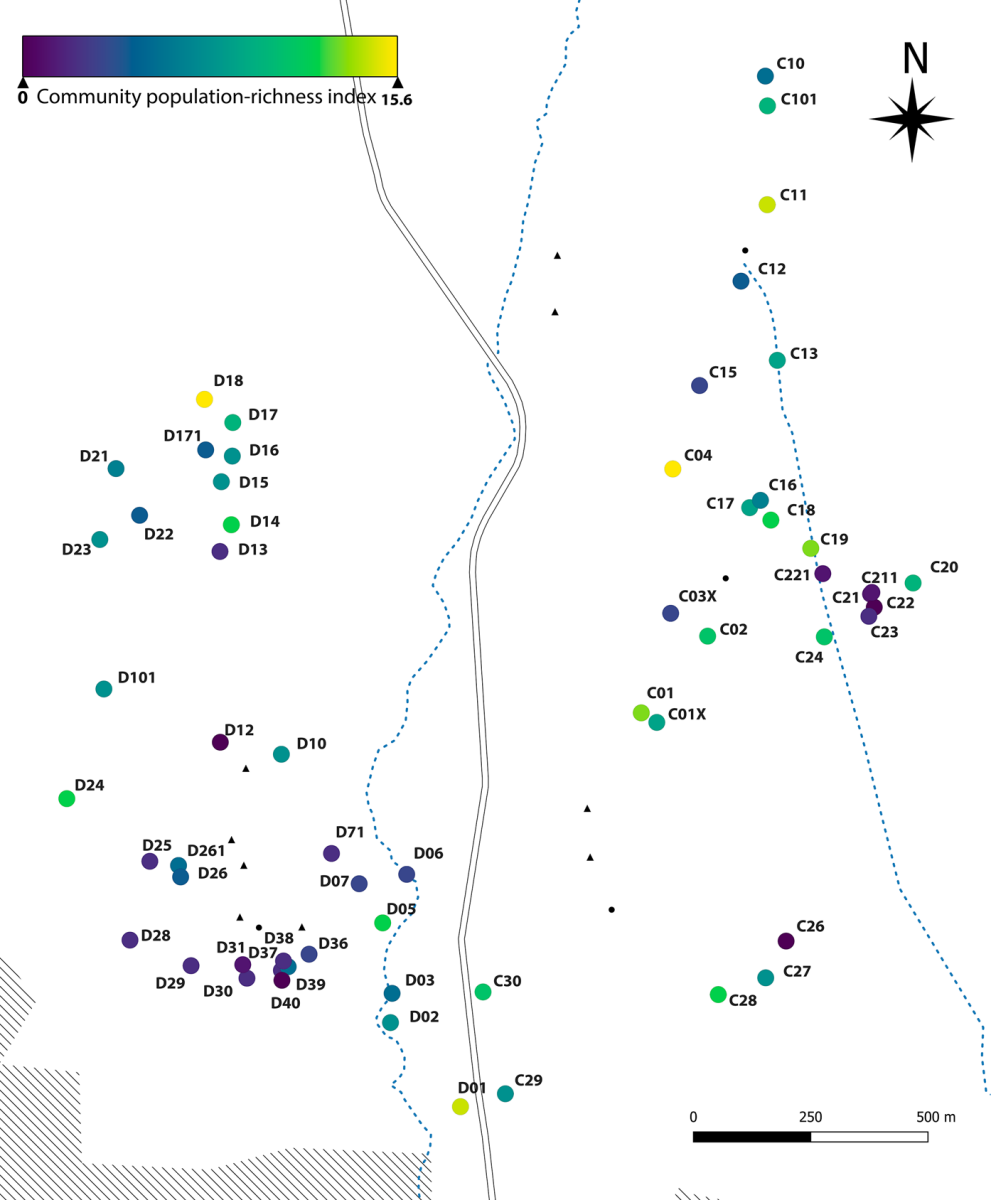
Community-level importance of ponds in the study area. For this map, population sizes of each species were scaled globally. In each pond, the scaled values for all species were then summed to obtain an index that accounts for both species richness and relative population sizes in each pond. Alphanumeric codes indicate pond IDs

Precise data on the use of ponds as stepping-stones is difficult to obtain with capture-recapture techniques. The relatively low dispersal frequency of amphibians and the presumable transience of the individuals in those ponds make these movements easy to miss. Apart from indirect evidence, such as single captures of individuals which had moved to or from very small ponds without breeding populations, we obtained direct evidence of the use of small ponds as stepping-stones in a female *P. perezi*. This individual was detected 3 times during 2 months in D15, a small northwestern pond (Fig. 3), then recaptured again 3 weeks later in the very small pond D22, which was probably used as a stepping-stone (no recaptures despite very easy detection), to reach the small pond D23, where she was recaptured the next month and again 9 days later. She was detected for the last time in the permanent pond D16 40 days later. The cumulative distance covered by this individual was 512 m.

### Dispersal kernels

**Fig. 4:**
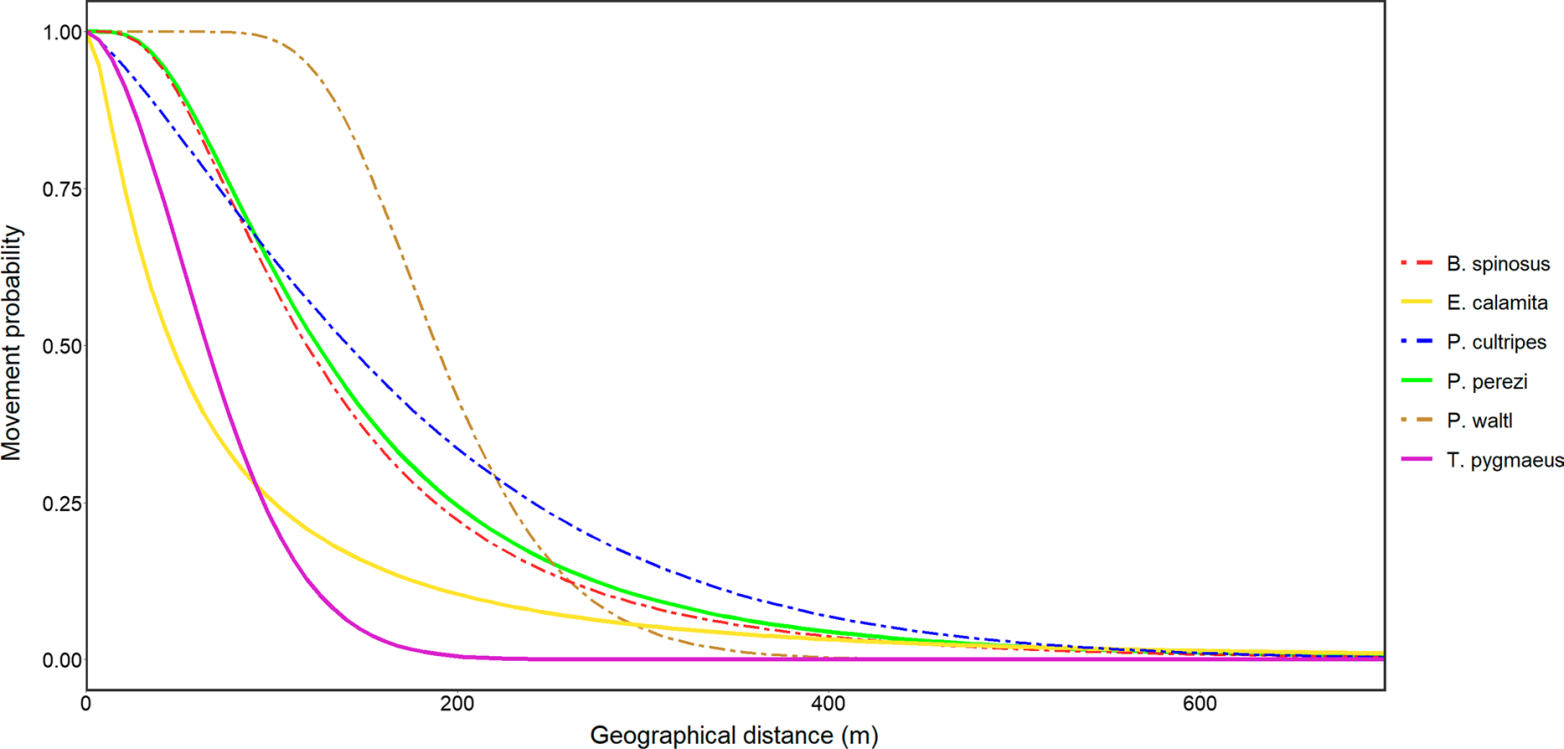
Dispersal kernels fitted for six amphibian species in the study area. Dashed lines represent species for which recorded movements were scarce and distribution model selection was inconclusive

Weibull was selected as the best fitting distribution for dispersal kernels in *P. cultripes* (ΔAIC = 0.37) and *T. pygmaeus* (ΔAIC = 2.75), whereas lognormal was the best fitting for *P. perezi* (ΔAIC = 37.14), *E. calamita* (ΔAIC = 15.65), *P. waltl* (ΔAIC = 1.3), and *B. spinosus* (ΔAIC = 1.24). In some cases, selection of the best-fitting distribution was inconclusive (ΔAIC < 2) due to the scarcity of movement data, namely in *P. cultripes*, *P. waltl*, and *B. spinosus*. Dispersal kernels are illustrated in Fig. 4.

### Abundance

We were able to perform all R2ucare GOF tests only in the largest populations; in smaller ponds, the low number of captures and/or the structure of the data precluded use of GOF tests. Regardless, we did not find any evidence of consistent GOF deviations (Online Resource 3). Model selection data and the estimated population sizes can be found in the supplementary material (Online Resource 4 and 5). We found marked differences in population size between ponds for all species (Online Resource 1: Figs. S4 and S5). The most populous ponds were generally reproductive ponds, and, usually, those where reproduction was recorded in both years. For the most aquatic species (*P. perezi, P. waltl* and *T. pygmaeus*, *L. boscai*) the majority of populations were concentrated in a few ponds. Some of these ponds, such as D01, D18 and C04 (Fig. 3) sustained large populations of at least three of these species (*P. perezi, P. waltl* and *T. pygmaeus*). These ponds did not usually coincide with the most populous pond of the more terrestrial species (*E. calamita*, *P. cultripes*, *B. spinosus*). Apart from these large reproductive ponds, the rest of the individuals of all species were distributed in a variable number of ponds that sustained small populations, and in ponds which were used temporarily as stepping-stones or were inhabited by just a few resident individuals.

### Pondscape graphs

**Fig. 5:**
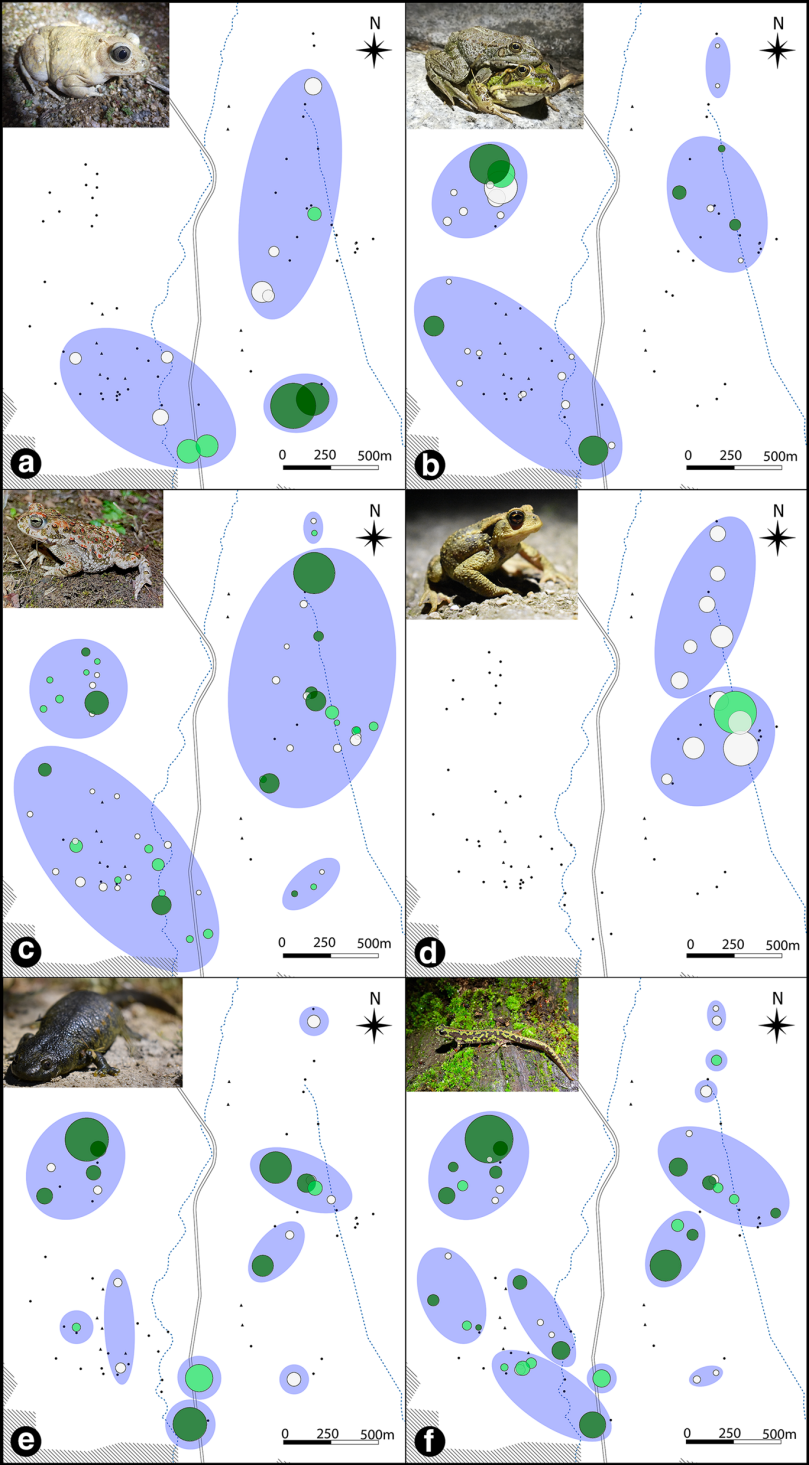
Map showing relative dPC_k_ values of the ponds for **A** *Pelobates cultripes*, **B** *Pelophylax perezi*, **C** *Epidalea calamita*, **D** *Bufo spinosus*, **E** *Pleurodeles waltl* and **F** *Triturus pygmaeus*. Node sizes are proportional to squared dPC_k_. The color of the nodes represents whether reproduction was registered in both years (dark green), 1 year (light green) or none (grey) for each species. Blue ellipses represent the clusters recovered by the walktrap method

Urodele graphs were generally more fragmentary, with 9 and 11 clusters for *P. waltl* and *T. pygmaeus* respectively, whereas for anurans, between 2 and 5 clusters per graph were identified (Fig. 5). Clustering showed some shared characteristics in the connectivity structure of most species. A northwestern cluster of ponds was identified for all species. The same was true for a small group of ponds in the southeasternmost part of the study area. The northeastern ponds were also identified as relatively isolated from the rest for several species. An extensive cluster in the southwest was identified for all anurans. Modularity values are available in the supplementary material (Online Resource 6).

dPC_k_ values varied among species (Fig. 5 and Online Resource 6). Important ponds were usually related to populous breeding ponds and their dPC_k_ value was strongly influenced by dPCflux_k_ and dPCintra_k_, while dPCconnector_k_ was very small for most species (even 0 in some of them).

**Fig. 6:**
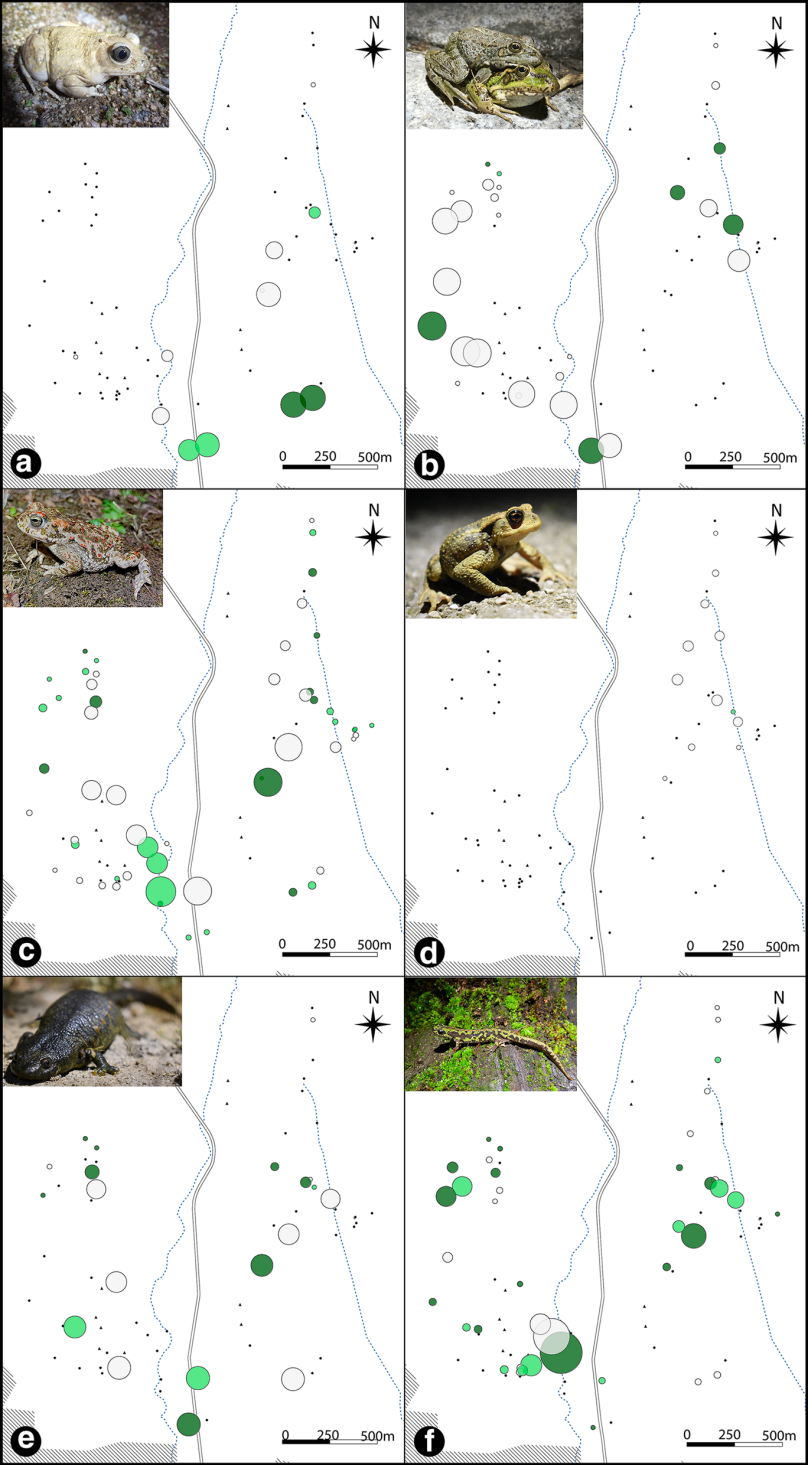
Map showing relative betweenness values of the ponds for **A** *Pelobates cultripes*, **B** *Pelophylax perezi*, **C** *Epidalea calamita*, **D** *Bufo spinosus*, **E** *Pleurodeles waltl* and **F** *Triturus pygmaeus*. Node sizes are proportional to betweenness. The color of the nodes represents whether reproduction was registered in both years (dark green), 1 year (light green) or none (grey) for each species

Betweenness values also varied for each species. The maps (Fig. 6) show different groups of ponds with high betweenness that condense the shortest paths of the whole graph for each species. Most of the species showed a high value of betweenness for some ponds located in the southern areas of the map, reflecting the position of the most likely path connecting the eastern and western sectors.

We found three groups of ponds with a relatively high value of community index for richness and population size (Fig. 3 and Online Resource 7). First, a northwestern group which hosts large populations of the most common species as well as an important reproductive pond for *H. molleri*. Second, a central eastern group with the most populous and interconnected ponds of the eastern sector, as well as the largest populations of *L. boscai*. And third, a southern group that comprises ponds from both sectors, the most favorable area for road crossing, important populations of common species and the only breeding ponds of the rarest species in the study site, *D. galganoi* and *A. cisternasii*; it is also the main breeding area of *P. cultripes* and includes a breeding pond for *H. molleri*. Outside of these major groups we found some isolated ponds with high index scores, such as D24 and C11, which host important populations of *P. perezi* and *E. calamita* respectively, as well as other syntopic species.

## Discussion

We present a novel approach to characterize fine-scale patterns of functional connectivity in amphibians within pondscapes that exploits individual photo-identification to estimate patterns of abundance and document key aspects of their spatial ecology. Our results allow the quantitative characterization of functional connectivity across a pond network and the identification of key nodes and links with the greatest contribution to overall (= metapopulation) connectivity.

### Photoidentification

The application of photoidentification of individuals was essential to obtain detailed data on the abundance and movement ecology of several species of Iberian pond-breeding amphibians for which there was little information available. These data set the basis for all subsequent network characterization, and the use of an accurate method of individual identification that did not affect the behavior of individuals was critical.

In the last decade, photoidentification has received increasing attention as a valuable alternative to more invasive methods for marking amphibians (Sannolo et al. 2016). The use of software designed to overcome the time-consuming labor of manual identification has further popularized this methodology (Van Tienhoven et al. 2007; Bolger et al. 2012; Moya et al. 2015). Overall, our results confirm that photoidentification is an effective, non-invasive and inexpensive method readily applicable for individual recognition in capture-recapture studies targeted to pond-breeding amphibian communities and that it is feasible despite coloration changes.

### Spatial ecology and structure of the amphibian community

In accordance with Cayuela et al. (2020), we found that movement frequency was much higher in anurans than in urodeles. Within anurans, *E. calamita*, *B. spinosus* and *P. cultripes*, which make an extensive use of the terrestrial habitat matrix (Gómez-Mestre 2014; Ortiz-Santaliestra 2014; Recuero 2014), showed higher movement frequencies than the more aquatic *P. perezi* (Egea-Serrano 2014). Overall patterns of breeding pond selection, observed dispersal distances and population connectivity can be explained in the context of the different ecological habits of the different species (Table 2).

With regard to species-specific movement distances, we only detected a significant difference between two species. *Epidalea calamita* had a lower average movement distance than *P. perezi*, which differed from our expectations given the highly terrestrial habits of *E. calamita* (Gómez-Mestre 2014). This species was more frequently detected in the terrestrial matrix than in ponds, resulting in shorter average movement distances than for *P. perezi*, which were almost exclusively detected in ponds, in accordance with their aquatic habits (Egea-Serrano 2014). Thus, recorded movement distances for *P. perezi* were limited to the longer geographic distances between pairs of ponds.

The movement data obtained during 2 years of monitoring allowed us to generate reliable kernels for at least two abundant species with comparatively high movement rates (*P. perezi* and *E. calamita*). For abundant species with lower movement frequencies (in this case, urodeles), while kernels served our purposes for graph modelling, they could be improved by extending the sampling period over additional breeding seasons. It should be noted that these species were also, coincidentally, those with a later onset of photoidentification, which reduced the number of potential movements to be detected. It should also be stressed that kernels reported here are specific to the habitat and climate of the study site and may vary across the range of individual species. Also, our study extended over two monitoring years. Kernels obtained over a more restricted time interval may reflect seasonal differences.

The lack of dispersal records in some species in our study can be explained by a low number of recaptures (*L. boscai*) or by their low abundance (*H. molleri* and *D. galganoi*), because the number of detected movements is often proportional to population size (Sullivan et al. 2021). For these species, reconstructing representative dispersal kernels requires specific surveys focused on their breeding sites and/or longer time series.

Our study zone is a “dehesa” landscape divided by a major road. Roads are generally regarded as potential barriers to amphibian movement (Gibbs 1998; Jochimsen et al. 2004; García-González et al. 2012). During our study, we fortuitously observed attempts by *E. calamita* to cross the road during nights with heavy rain, resulting in high mortality. Furthermore, we only detected a single road crossing event via recaptures in 2 years of monitoring (one individual of *P. cultripes*). This evidence suggests that road M-601 acts as a barrier for the dispersal of amphibians in the study area. We included the effect of the road in graphs as a length multiplier. While other options were tested, the results were very similar, with the south of the study area having the shortest distances between ponds separated by the road, and therefore affording the most probable route for road crossing, in contrast with the north, where distances between ponds on opposite sides of the road are long and fall into the asymptotic region of the dispersal kernels.

### Applications of population graphs

Probability graphs, such as those constructed in this study, provide valuable tools to model amphibian (meta)community dynamics. The structure of the network and the connectivity measurements inferred probabilistically from field data allow assessing the relative role of each node (pond) in maintaining functional connectivity in the population network, which is key to planning effective conservation actions.

The marked differences in population sizes among ponds, coupled with the low dispersal probabilities of some species (and the effect of multiplicative distances in these probabilities) made the highest dPC_k_ values highly dependent on population size (Online Resource 6). While this is expected, and ponds with large populations are indeed of the greatest importance, we should not dismiss the role of ponds with a much lower range of dPC_k_ (as proposed by Jordán et al. 2003 and Matos et al. 2019 for other connectivity measurements), especially for species such as *T. pygmaeus*, in which a single extremely populous pond takes up a large proportion of the total dPC_k_ given their high dPCintra_k_. We thus advise also exploring mid-range values of dPC_k_ and their fractions to account for ponds which, while not as populous, are placed high in the ranking for these indices relative to their population size (related to dPCintra_k_ and dPCflux_k_) or their role in the connectivity of the network (related to dPCflux_k_ and dPCconnector_k_). In addition, we also advise assessing the importance of ponds for stepping-stone dispersal using betweenness values.

The number of clusters provides a quantification of relative network fragmentation. A high number of clusters indicates that a network is composed of groups of highly connected populations (or isolated ponds) joined by relatively weak connections with a much lower frequency of migrants among them. Overall, urodele populations were characterized by more fragmentary networks than anurans, but this pattern was not necessarily related to differences in life history traits specific to this clade. The fragmentation of the *T. pygmaeus* network can be explained by shorter dispersal distances, while in the case of *P. waltl*, fragmentation seemed to be caused by a larger geographical separation of the populated ponds. This could be related to differences in habitat preferences, as *P. waltl* was mostly found in relatively large and deep ponds with extended hydroperiods, while *T. pygmaeus* was present in ponds with a broader variety of sizes and hydroperiods, in accordance with the literature on their breeding site preferences (Table 2). The differences between the clusters found for anurans may be explained by their distribution: the clusters of *P. perezi* and *E. calamita* are very similar, except for the absence of *P. perezi* in the southeast. The reduced number of clusters in *P. cultripes* is influenced by the lack of individuals associated with ponds in the northwest. *Bufo spinosus* showed the lowest modularity and cluster count, with only two clusters. This was expected due to its restricted distribution in our study site, consisting of a small group of ponds close to each other.

The results of this study are based solely on movement data for adults because of the difficulty of tracking juvenile amphibians. The contribution of natal dispersal (the dispersal of juveniles from their natal pond to a reproductive pond) to the overall dispersal is undoubtedly important and likely varies among species and populations (Cayuela et al. 2020). The potential contribution of dispersing juveniles to population connectivity could be accounted for with complementary studies on the genetic structure of amphibian populations.

## Conclusion

The combination of photoidentification, capture-recapture techniques and graph theory allowed us to finely characterize pond-breeding amphibian metapopulations at a community scale. The fine-scale movement and population data allowed the for generation of graphs that quantified functional connectivity and the most probable dispersal routes, as well as the least likely connections. The resulting networks represent a powerful tool to quantitatively assess node importance and connection probability, which are key for designing evidence-based management decisions for pond restoration or improvement of landscape connectivity. In the case of pondscapes, this approach can be used to determine where to create new ponds or to prioritize ponds that have a higher contribution to connectivity for conservation and management, as well as to identify locations where amphibian road crossing infrastructure could enhance connectivity. Lastly, the contribution of individual ponds to the functional connectivity of the network varied by species, due to differences in life history and movement ecology, highlighting the need to account for interspecific differences in the study of amphibian pondscapes.

**Table 1:** Capture and movement data for all ponds and amphibian species of the study area.

	PP	EC	PC	BS	HM	DG	AC	PW	TP	LB
Total number of different individuals encountered (2019–2020)	456	1725	58	49	27	6	0	243	1817	155
Individuals encountered on the western sector (2019–2020)	387	644	14	0	27	6	0	129	1255	26
Individuals encountered on the eastern sector (2019–2020)	69	1081	44	49	0	0	Larvae	114	562	129
Total number of recaptures (2019–2020)	712	189	23	17	23	2	0	329	1187	80
Number of recaptures identified by PhotoID (2019–2020)	712	189	23	17	23	2	0	291	652	46
Proportion of individuals recaptured at least once (2019–2020)	0.6	0.09	0.27	0.24	0.48	0.17	0	0.52	0.25	0.19
Number (proportion) of used ponds (2019–2020)	28 (0.44)	55 (0.86)	12 (0.19)	12 (0.19)	5 (0.08)	2 (0.03)	1 (0.02)	20 (0.31)	40 (0.63)	17 (0.27)
Proportion of used ponds with breeding activity (2019–2020)	0.28	0.56	0.42	0.08	0.6	0.5	1	0.63	0.63	0.18
Number of encounters on the terrestrial matrix (2019–2020)	3	725	12	20	0	1	0	1	3	0
Number of dispersal movements recorded (2019–2021)	104	74	9	8	0	0	0	6	12	0
Number of dispersal movements recorded (2019–2020)	101	74	9	8	0	0	0	6	8	0
Maximum single movement distance (m) (2019–2021)	910	880	420	350	NA	NA	NA	280	150	NA
Average movement distance from raw data (m) (2019–2021)	163.96	94.53	172.56	150.75	NA	NA	NA	196.67	71.08	NA
Median movement distance from raw data (m) (2019–2021)	130	45	120	99	NA	NA	NA	160	75	NA
Maximum cumulated distance traveled by a single individual (m) (2019–2021)	910	880	477	350	NA	NA	NA	560	150	NA
Movement frequency (2019–2020)	0.14	0.39	0.39	0.47	0	0	NA	0.02	0.01	0

Values related to dispersal distance use data from 2021 in addition to that of 2019–2020. PP *Pelophylax perezi*, EC *Epidalea calamita*, PC *Pelobates cultripes*, BS *Bufo spinosus*, HM *Hyla molleri*, DG *Discoglossus galganoi*, AC *Alytes cisternasii*, PW *Pleurodeles waltl*, TP *Triturus pygmaeus*, LB *Lissotriton boscai*

**Table 2:** Ecological characteristics of the six amphibian species included in graph analyses. For species categorized as “terrestrial” in the first column, the second column refers mostly to reproductive pond preferences. References: Egea-Serrano (2014) (*P. perezi*), Gomez-Mestre (2014) (*E. calamita*), Ortiz-Santaliestra (2014) (*B. spinosus*), Recuero (2014) (*P. cultripes*), Reques (2014) (*T. pygmaeus*) and Salvador (2014) (*P. waltl*)

	Predominant activity	Pond preferences	Maximum altitude	Vegetation preferences	Inactivity period	Terrestrial phase
*Pelophylax perezi*	aquatic	Wide range of characteristics. Vegetated shores. Reproduction in deep ponds	2380 m	Ponds with vegetated shores	Reduced activity during the coldest months	–
*Epidalea calamita*	terrestrial	Temporary shallow ponds	> 2500 m	Open areas with low and sparse vegetation	Buries underground	–
*Pelobates cultripes*	terrestrial	Long hydroperiod	1770 m	No clear preference	Buries underground in uncompacted or sandy soil	–
*Bufo spinosus*	terrestrial	Deep ponds with long hydroperiod, aquatic vegetation	2600 m	No clear preference	In rodent galleries or holes under trees	–
*Pleurodeles waltl*	aquatic	Large deep ponds with long hydroperiod, clay substrate	1480 m	Presence positively correlated with “dehesas”	Stays in water or buries underground	Little known, mostly in refuges, terrestrial activity reduced with low temperatures
*Triturus pygmaeus*	aquatic	Wide range of characteristics, abundant aquatic vegetation	1500 m	No clear preference	Underground, under rocks and tree roots	Little known, mostly found in refuges

## Electronic supplementary material

Below is the link to the electronic supplementary material.

Supplementary Material 1

Supplementary Material 2

Supplementary Material 3

Supplementary Material 4

Supplementary Material 5

Supplementary Material 6

Supplementary Material 7

## Data Availability

The datasets generated during the current study are available in the repository 10.6084/m9.figshare.21158608.
