# Supplementary material for: Integrating dispersal, breeding and abundance data with graph theory for the characterization and management of functional connectivity in amphibian pondscapes
Source: Landsc Ecol. 2022 Nov 3;37(12):3159–77. doi: 10.1007/s10980-022-01520-x (PMC9631601; doi:10.1007/s10980-022-01520-x)
Supplement: Supplementary file 1 — Supplementary Material 1 [file 10980_2022_1520_MOESM1_ESM.pdf]

Online Resource 1

Integrating dispersal, breeding and abundance data with graph theory for the characterization and management of functional connectivity in amphibian pondscapes.

Ismael Reyes-Moya[1*], Gregorio Sánchez-Montes[2] & Íñigo Martínez-Solano[3]

Departamento de Biodiversidad y Biología Evolutiva, Museo Nacional de Ciencias Naturales (MNCN-CSIC), c/ José Gutiérrez Abascal 2, 28006 Madrid, Spain.

* Corresponding author: Ismael Reyes Moya. E-mail: ismaelrymy@gmail.com. Tlf: +34914111328

Landscape Ecology

This document contains supplementary figures concerning methodology and results of the paper.

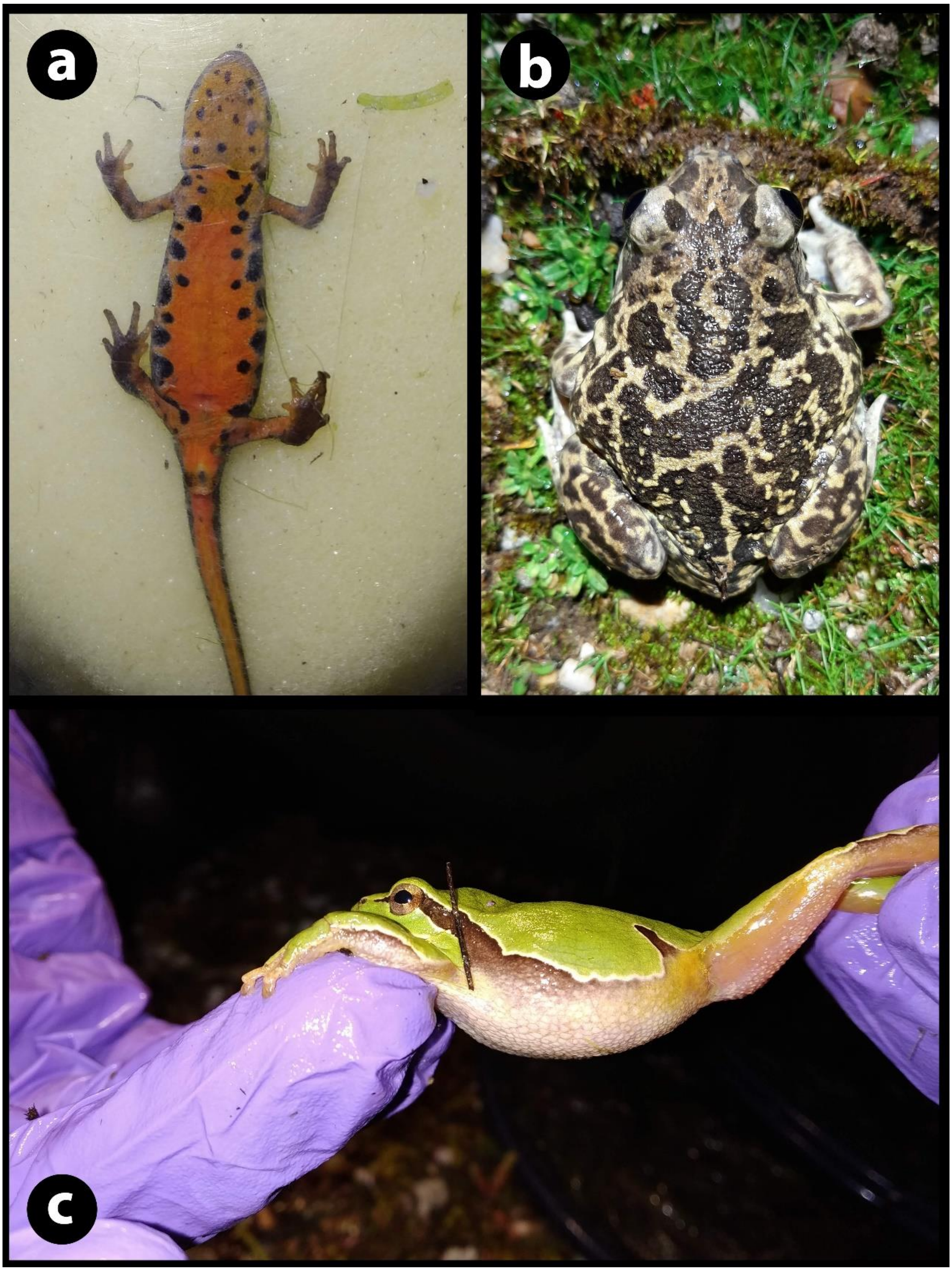

Fig S1 Photos showing the three methods used to identify individuals. a) Ventral photos (urodeles). b) Dorsal photos (anurans). c) Lateral photos (H. molleri).

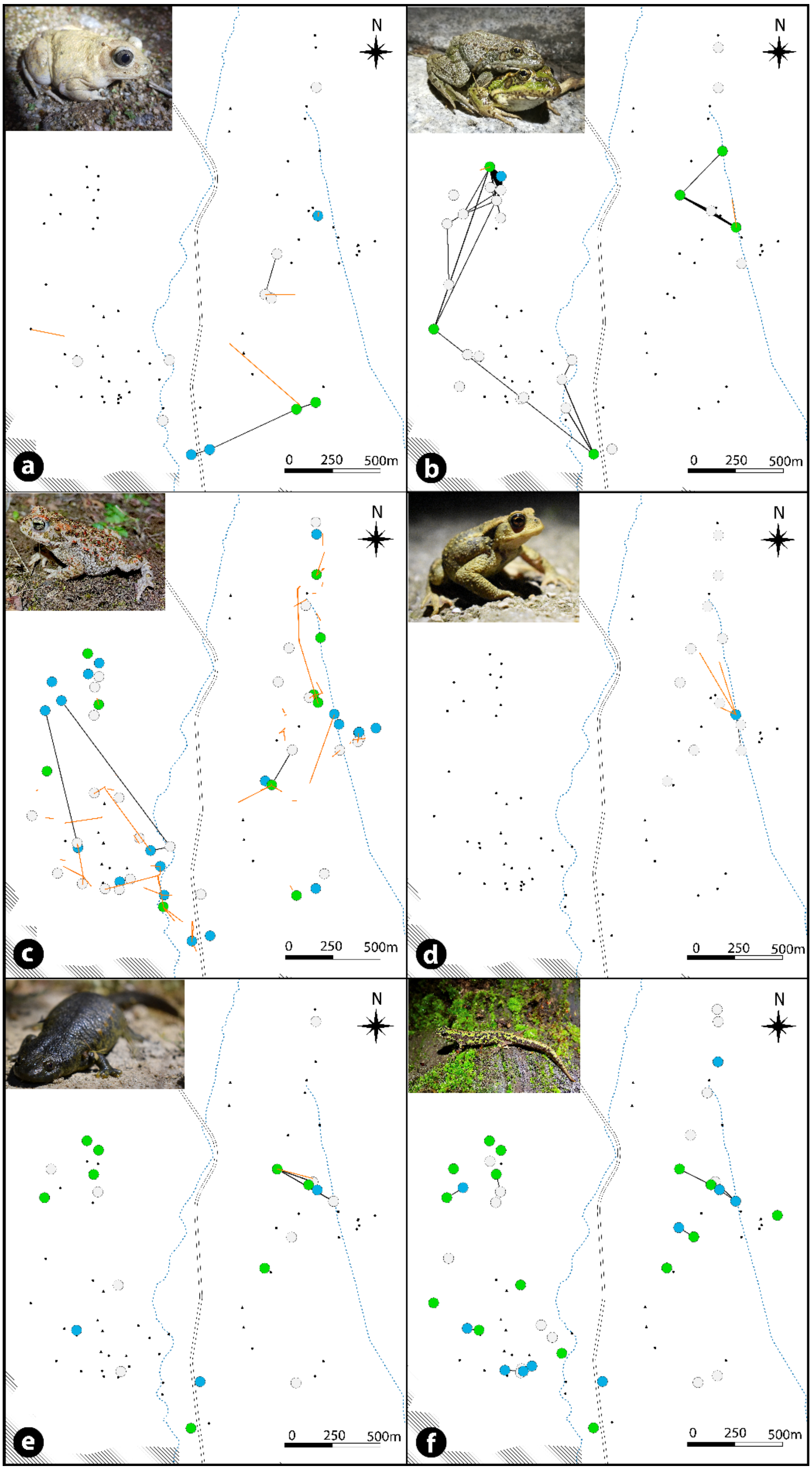

Fig S2 Maps showing the detected movements of a) Pelobates cultripes, b) Pelophylax perezi, c) Epidalea calamita, d) Bufo spinosus, e) Pleurodeles waltl and f) Triturus pygmaeus. Trajectories of movements involving the terrestrial matrix are marked in orange. The color of the nodes represents whether reproduction was registered in one year (blue), both years (green) or none (grey) for each species. Line thickness is proportional to the number of detected movements in each species.

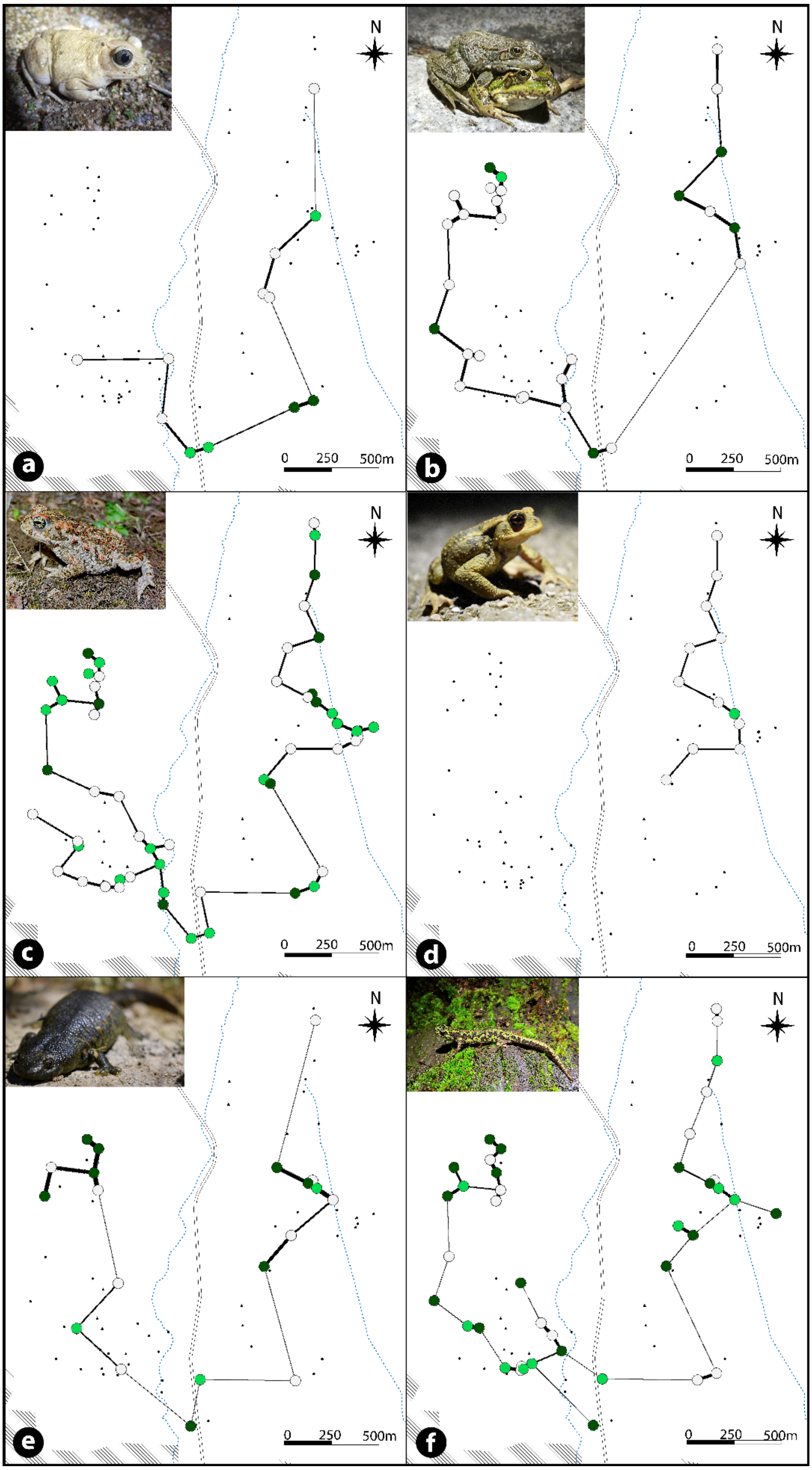

Fig S3 Maps showing the Minimum Spanning Trees of a) Pelobates cultripes, b) Pelophylax perezi, c) Epidalea calamita, d) Bufo spinosus, e) Pleurodeles waltl and f) Triturus pygmaeus. The color of the nodes represents whether reproduction was registered in both years (dark green), one year (light green) or none (grey) for each species. Line thickness is proportional to the probability of connection.

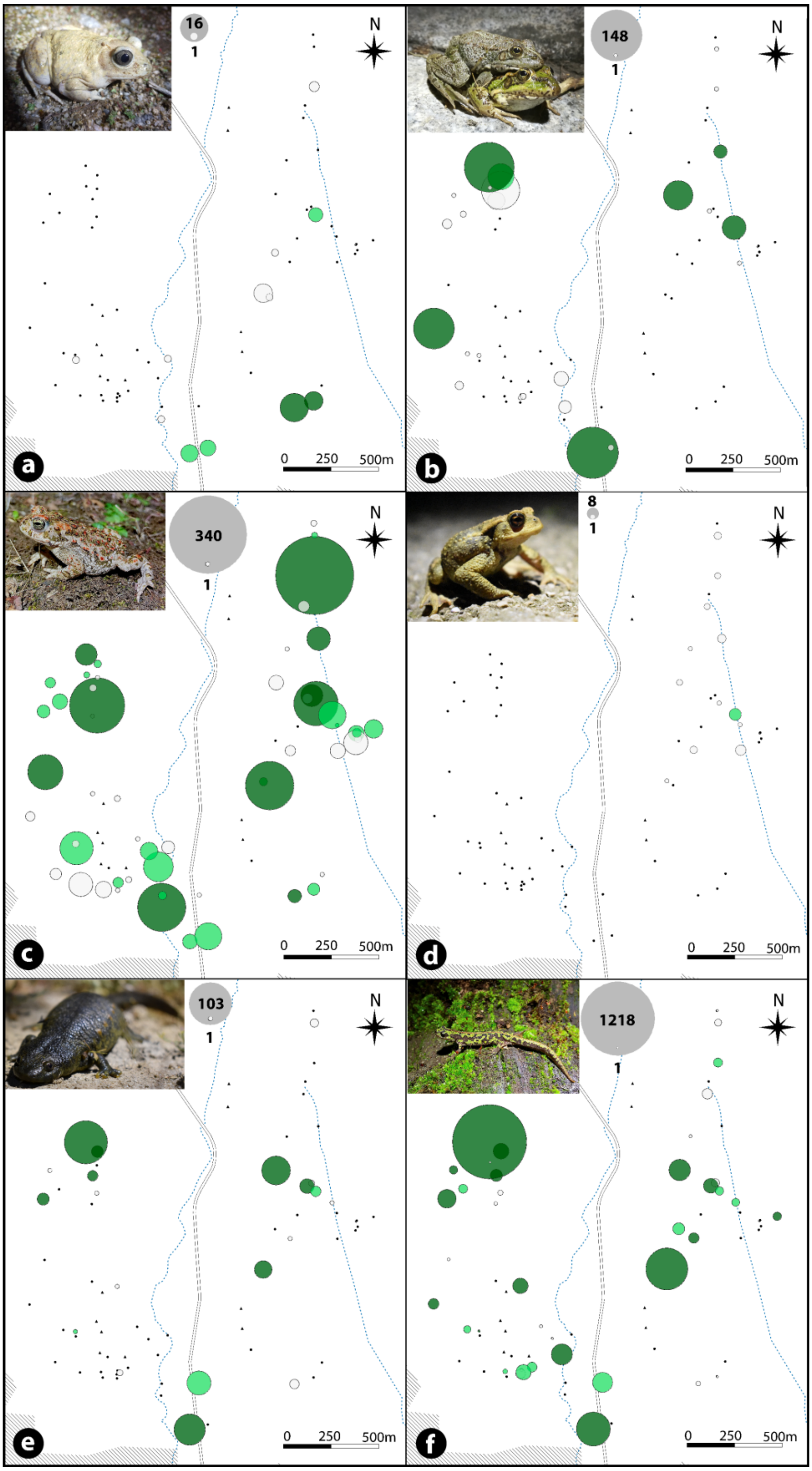

Fig S4 Maps showing the estimated population sizes of a) Pelobates cultripes, b) Pelophylax perezi, c) Epidalea calamita, d) Bufo spinosus, e) Pleurodeles waltl and f) Triturus pygmaeus. The color of the nodes represents whether reproduction was registered in both years (dark green), one year (light green) or none (grey) for each species. Node size is proportional to population size. Minimum and maximum population sizes and their respective node sizes are provided in each map.

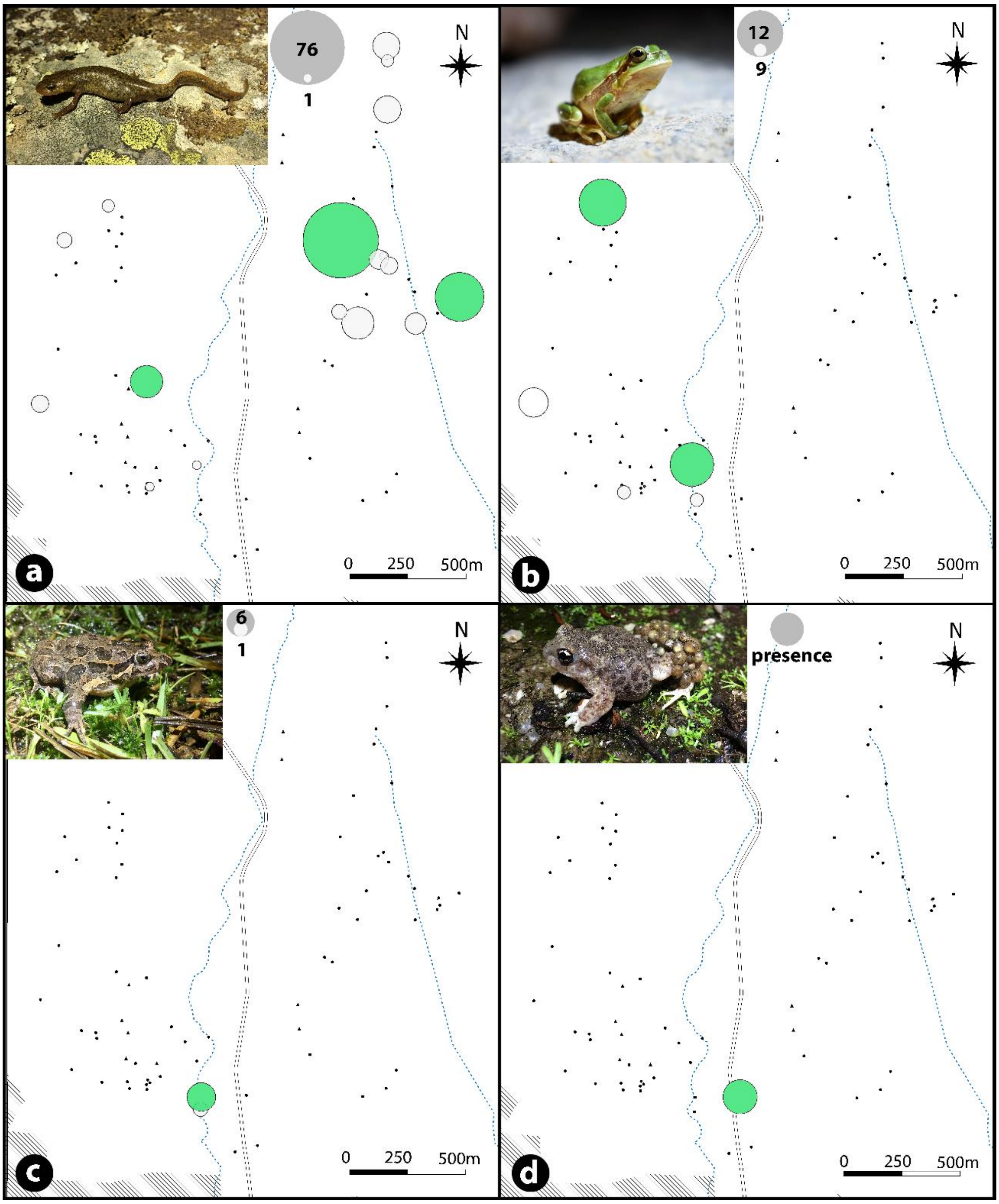

Fig S5 Maps showing the estimated population sizes of a) Lissotriton boscai, b) Hyla molleri, c) Discoglossus galganoi and d) Alytes cisternasii. The color of the nodes represents whether reproduction was registered (green) or not (grey) for each species. Node size is proportional to population size, except for A. cisternasii, for which node size is irrelevant, as only presence is represented. Minimum and maximum population sizes and their respective node sizes are provided in each map.
