# Supplementary material for: Integrating dispersal, breeding and abundance data with graph theory for the characterization and management of functional connectivity in amphibian pondscapes
Source: Landsc Ecol. 2022 Nov 3;37(12):3159–77. doi: 10.1007/s10980-022-01520-x (PMC9631601; doi:10.1007/s10980-022-01520-x)
Supplement: Supplementary file 2 — Supplementary Material 2 [file 10980_2022_1520_MOESM2_ESM.pdf]

Online Resource 2

Integrating dispersal, breeding and abundance data with graph theory for the characterization and management of functional connectivity in amphibian pondscapes.

Ismael Reyes-Moya[1*], Gregorio Sánchez-Montes[2] & Íñigo Martínez-Solano[3]

Departamento de Biodiversidad y Biología Evolutiva, Museo Nacional de Ciencias Naturales (MNCN-CSIC), c/ José Gutiérrez Abascal 2, 28006 Madrid, Spain.

* Corresponding author: Ismael Reyes Moya. E-mail: ismaelrymy@gmail.com. Tlf: +34914111328

Landscape Ecology

This document contains supplementary information and images concerning color changes detected during photoidentification.

COLOR CHANGES

Color changes difficulted manual photoidentification of several species. We detected base color changes (*P. perezi*, *E. calamita*), dorsal midline color changes (*P. perezi*), dorsal midline fading (*P. perezi*), pattern fading, principally in the dorsal area (*P. perezi*), belly spots contraction/expansion (*T. pygmaeus*) and belly darkening (*P. waltl*) (Fig. S5). Some less striking color changes were detected in other species such as *B. spinosus*, *P. cultripes* and *L. boscai*. Changes in skin hydration and wart volume sometimes made the identification of individuals of *B. spinosus* and *E. calamita* challenging.

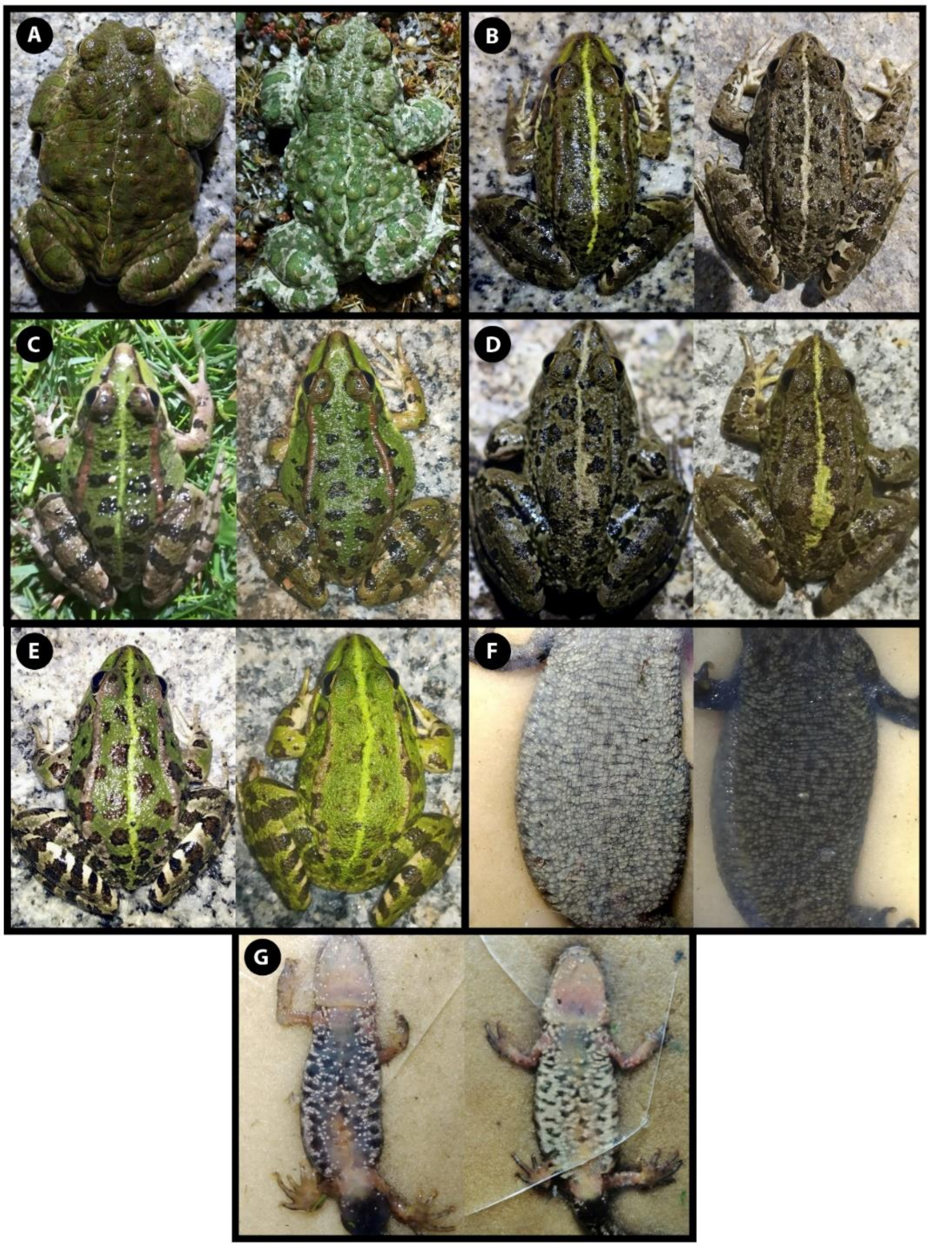

Fig S1 Examples of color changes detected in recaptured individuals. A, B: Base color changes in *E. calamita* and *P. perezi*, respectively. C, D: Dorsal midline fading and color change, respectively, in *P. perezi*. E: Pattern fading in *P. perezi*. F: Underside darkening in *P. waltl*. F: White spots expansion/contraction in *T. pygmaeus*.
